# Computational analysis of the oscillatory behavior at the translation level induced by mRNA levels oscillations due to finite intracellular resources

**DOI:** 10.1371/journal.pcbi.1006055

**Published:** 2018-04-03

**Authors:** Yoram Zarai, Tamir Tuller

**Affiliations:** 1 Department of Biomedical Engineering, Tel-Aviv University, Tel-Aviv, Israel; 2 Department of Biomedical Engineering and the Sagol School of Neuroscience, Tel-Aviv University, Tel-Aviv, Israel; Rutgers University, UNITED STATES

## Abstract

Recent studies have demonstrated how the competition for the finite pool of available gene expression factors has important effect on fundamental gene expression aspects. In this study, based on a whole-cell model simulation of translation in *S. cerevisiae*, we evaluate for the first time the expected effect of mRNA levels fluctuations on translation due to the finite pool of ribosomes. We show that fluctuations of a single gene or a group of genes mRNA levels induce periodic behavior in all *S. cerevisiae* translation factors and aspects: the ribosomal densities and the translation rates of all *S. cerevisiae* mRNAs oscillate. We numerically measure the oscillation amplitudes demonstrating that fluctuations of endogenous and heterologous genes can cause a significant fluctuation of up to 50% in the steady-state translation rates of the rest of the genes. Furthermore, we demonstrate by synonymous mutations that oscillating the levels of mRNAs that experience high ribosomal occupancy (e.g. ribosomal “traffic jam”) induces the largest impact on the translation of the *S. cerevisiae* genome.

The results reported here should provide novel insights and principles related to the design of synthetic gene expression circuits and related to the evolutionary constraints shaping gene expression of endogenous genes.

## Author summary

Each cell contains a limited number of macromolecules and factors that participate in the gene expression process. These expression resources are shared between the different molecules that encode the genetic code, resulting in non-trivial couplings and competitions between the different gene expression stages. Such competitions should be considered when analyzing the cellular economy of the cell, the genome evolution, and the design of synthetic expression circuits. Here we study the effect of couplings and competitions for ribosomes by performing a whole-cell simulation of translation of *S. cerevisiae*, with parameters estimated from experimental data. We demonstrate that by periodically changing the mRNA levels of a single gene (endogenous or heterologous) or a set of genes, the translation of all *S. cerevisiae* genes are affected in a periodic manner. We numerically estimate the exact impact of the mRNA levels periodicity on the translation process dynamics, as well as on the dynamics of the free ribosomal pool and the way it is affected by parameters such as the codon composition of the oscillating gene, its initiation rate and mRNA levels. Furthermore, we show that the codon compositions of synthetically highly expressed heterologous genes that are expected to oscillate must be carefully considered. For example, synonymous mutations resulting in “traffic jams” of ribosomes along the fluctuated mRNAs may cause significant fluctuations of up to 50% in the steady-state translation rates of all genes.

## Introduction

During the gene expression process various macromolecules (e.g. ribosomes, RNA polymerase (RNAP), transcription factors, elongation factors, spliceosome, transfer RNA (tRNA) molecules, etc.) process the genetic material (DNA, mRNA, pre-mRNA) in order to generate proteins [1]. The number of gene expression macromolecules and factors in the cell is finite; for example, there are about 200,000 ribosomes and 30,000 RNAP-II molecules in the *S. cerevisiae* cell [2, 3]. Thus, this limited resource budget induces competition between the different molecules/regions encoding the genetic material, resulting in non-trivial correlations and couplings between the different gene expression stages, and between the processed genetic material molecules.

Some previous studies have suggested that such competition should be considered when designing synthetic gene expression circuits [4–9], and that they significantly affect the evolution of genomes [10]. For example, [9] considered a stochastic model to analyze the competition of two types of mRNAs (two genes) for the limited ribosomal resource, where the total number of mRNAs and ribosomes fluctuate randomly. It was shown that the strength of the couplings (or cross-talk) between the translation of the two protein types strongly depends on whether the ribosomes are underloaded (i.e., there are more ribosomes than mRNAs) or overloaded (i.e., there are more mRNAs than ribosomes).

Specifically, it was also suggested that the competition for the limited main resources in transcription (RNAP [11]) and translation (ribosomes [12]) is a primary factor in the cellular economy of the cell. The competition for the available resources, which leads to an indirect coupling between expressions of different genes, might be one of the reasons why levels of genes, mRNAs, and proteins in the cell do not necessarily correlate [4, 10, 12–16].

The expression levels of large sets of genes and relevant gene expression factors are fluctuating or oscillating in different physiological conditions (e.g. cell cycle [17–21]). In addition, there are many cases of oscillating genes that are significant (up to hundreds of genes oscillating with a ratio of up to about three folds between highest to lowest mRNA levels) in all domains of life [22–34]. Furtheremore, various synthetic circuits and cell free systems include oscillators [35–42]. The couplings, due to competition, may link the oscillations related to one gene expression stage (e.g. transcription) to oscillations in a different gene expression stage (e.g. translation). In this study, we suggest for the first time that finite intracellular resources induce non-trivial and significant coupling between different gene expression stages (transcription and translation) in endogenous and heterologous genes. For example, increased mRNA levels in one gene affects the translation levels of all other genes. To this end, we perform a whole-cell simulation of translation [43, 44], which captures fundamental properties of translation, with parameters estimated from experimental data that enables us to comprehensively quantify these effects for the first time. This type of information is currently not available experimentally, and we believe that our results are expected to reflect well the reality.

We specifically demonstrate by Monte Carlo simulations that by periodically changing the mRNA levels of a single gene or a set of genes, i.e. by periodically modifying the transcription process, the translation of *all* *S. cerevisiae* genes are affected in a periodic manner, with the same periodicity as the mRNA levels periodicity. Importantly, we numerically estimate, for the first time, the exact impact of the mRNA levels periodicity on the translation process dynamics, as well as on the dynamics of the free ribosomal pool and the way it is affected by parameters such as the codon composition of the oscillating gene, its initiation rate and mRNA levels.

## Results and discussion

We utilize a large-scale, whole-cell computational model for simultaneous mRNA translation and competition for ribosomes to study the effect of mRNA levels fluctuation on the translation process [43–45]. The model considers all the fundamental properties of translation such as the different decoding times of codons and their order, the excluded volume interactions between ribosomes, the finite pool of ribosomes shared by all mRNAs, initiation rates, etc. [43–45]. The dynamics in this model is expressed by a set of ordinary differential equations describing the time evolution of the ribosomal occupancies in the different positions along the mRNAs, and the time evolution of the free ribosomal pool. It was shown that this computational model provides predictions with high correlation with protein levels and ribosome density measurements (see more details in the Materials and methods section).

We use the model to simulate translation of the *S. cerevisiae* genome including a heterologous green fluorescent protein (GFP) gene (with different codons compositions), while periodically modifying the mRNA levels of the GFP gene or of a subset of the endogenous genes. The competition for the limited, shared ribosomes, results in an indirect coupling between the translation processes of the different genes. We measure the coupling effect on the free pool of ribosomes, and on the translation rates and ribosomal densities of the different translation process as a function of the oscillating mRNA (or mRNAs) parameters and mutations.

**Fig 1:**
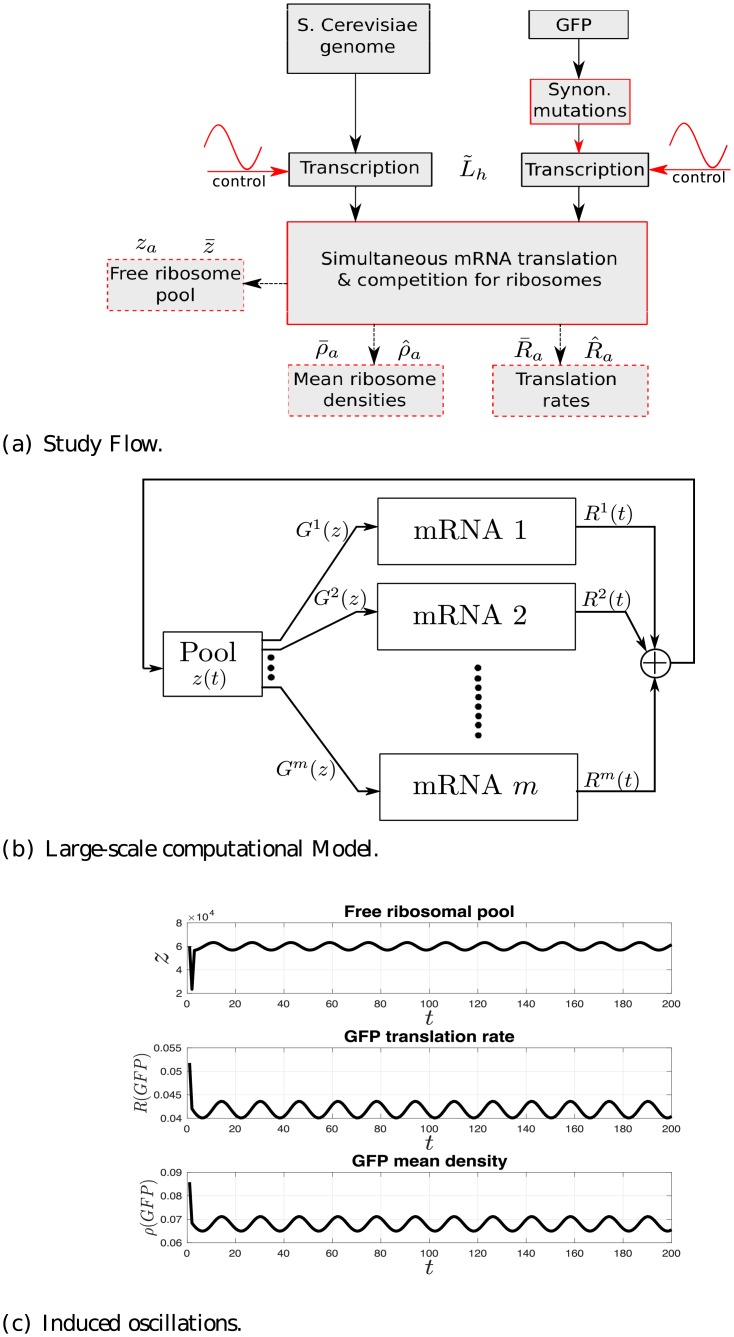
Overview of the study and the model. (a) Study flow diagram. Red color indicates blocks/functions simulated in this study. Dashed blocks specify measurements. Variables correspond to mRNA levels (${\tilde{L}}_{h}$) and various measurements ($z_{a},\bar{z},{\bar{R}}_{a},{\hat{R}}_{a},{\bar{\rho }}_{a},{\hat{\rho }}_{a}$). See the main text for details. (b) Topology of the computational model used in this study, which includes *m* mRNAs and a shared free pool of ribosomes. *G*^*i*^(*z*) denotes the initiation rate to mRNA *i*, and *R*^*i*^(*t*) denotes the translation rate of mRNA *i* at time *t*. (c) An example of the free ribosomal pool (top), the GFP translation rate (middle) and the GFP mean ribosomal density (bottom) as a function of *t* ∈ [0, 192] for a periodically varying GFP mRNA levels with period *T* = 16.

Fig 1(a) depicts our study flow diagram. We consider the *S. cerevisiae* genome and a GFP gene, while periodically controlling the transcription (i.e. the mRNA levels) of one or more *S. cerevisiae* genes or the GFP gene. All mRNAs are then simultaneous translated, while competing for the ribosomal resource. We then measure different translation parameters, such as translation rate and ribosomal density of all genes, and the free ribosomal pool (i.e. the number of free ribosomes). The block diagram of the model we use for simultaneous translation and competition is depicted in Fig 1(b). Please refer to the Materials and methods section for a detailed description of the model. Finally, Fig 1(c) shows an example of the translation parameters behavior when periodically controlling the mRNA levels of the GFP gene. The figure depicts the free ribosomal pool, and the GFP translation rate and mean ribosomal density as a function of time. It may be observed that these oscillate with a common periodicity.

It is important to mention that all the parameters used in the computational model (e.g. codon compositions, codon decoding times, mRNA levels, and number of ribosomes) were inferred based on experimental measurements of *S. cerevisiae*, and based on known properties of the GFP. Specifically, the *S. cerevisiae* genome consists of *m* ≔ 6310 protein-encoding genes with ORFs ranging from as low as 25 codons to as high as 4911 codons (see S4 Fig). The GFP gene ORF consists of 240 codons (see more details in the Materials and methods section).

Table 1 lists the parameters used throughout the simulations and their source.

**Table 1:** Computational model parameters.

Parameter	Value	Source
Total Ribosomal pool	200, 000	[2]
Total mRNA pool	60, 000	[46]
Median initiation rate	0.8 mRNA/sec	[47]
Median codon decoding rate	6.4 aa/sec	[48]

Let *L*_*h*_ denote the oscillating gene nominal mRNA levels and *α* its initiation rate. Recall that the oscillating gene (or genes) can be either an endogenous gene or the GFP heterologous gene. We periodically change the mRNA levels of the oscillating gene as follows:

$$l_{h}\left(t\right)=L_{h}(1+A\;\text{sin}\left(\frac{2\pi t}{T}\right)),$$ (1)

where ℓ_*h*_(*t*) denotes the oscillating gene mRNA levels at time *t*, *A* ∈ [0, 1) is the normalized amplitude, and *T* is the period time. Let *L*_*T*_ denote the total number of (*S. cerevisiae*) mRNAs in the cell (i.e. *L*_*T*_ ≔ 60, 000), and

$$\begin{array}{c} {\tilde{L}}_{h}≔100\frac{L_{h}}{L_{T}}, \end{array}$$

the oscillating gene nominal mRNA levels in percentage of *L*_*T*_.

Let *R*^*i*^ denote the average steady-state translation rate of gene *i*, $R_{amp}^{i}$ denote its steady-state amplitude, and

$$\begin{array}{c} R_{a}^{i}≔100\frac{R_{amp}^{i}}{R^{i}}, \end{array}$$

denote the steady-state translation rate amplitude of gene *i* in percentage of its average steady-state translation rate *R*^*i*^. Let

$$\begin{array}{c} {\bar{R}}_{a}≔\frac{\sum_{i=1}^{m}R_{a}^{i}}{m}, \end{array}$$

denote the average (over all genes) steady-state translation rate amplitude (in percentage).

In the same manner let (see Fig 1(a))

- $\rho_{a}^{i}$ denote the steady-state mean density amplitude of gene *i* in percentage of its average steady-state mean density *ρ*^*i*^,
- ${\bar{\rho }}_{a}$ denote the average (over all genes) steady-state mean density amplitude, in percentage of the average steady-state mean density,
- ${\hat{R}}_{a}$ denote the variance (over all genes) of the steady-state translation rate amplitude, in percentage of the average steady-state translation rate,
- ${\hat{\rho }}_{a}$ denote the variance (over all genes) of the steady-state mean density amplitude, in percentage of the average steady-state mean density,
- $\bar{z}$ denote the average free ribosomal pool at steady-state in percentage of the total ribosomal pool, and
- *z*_*a*_ denote the steady-state free ribosomal pool amplitude, in percentage of the average steady-state free pool.

See an example of these parameters in S5 Fig. The variance parameters provide indication of how the individual genes amplitude vary relative to the average. A large [small] variance implies that the genes amplitudes are widely [closely] scatter relative to the average.

As the intent in this paper is to analyze the impact of oscillations on the translation process, due to the shared, limited ribosomal resource, we believe that quantifying the above parameters under different conditions is essential in understanding the impact on the translation process.

In the following sections we present numeric measurements of the impact of periodically modifying the mRNA levels of endogenous genes or the GFP gene (or its mutations) on the above parameters over all the *S. cerevisiae* genes.

### The effect of endogenous gene mRNA levels on translation rate oscillations

**Fig 2:**
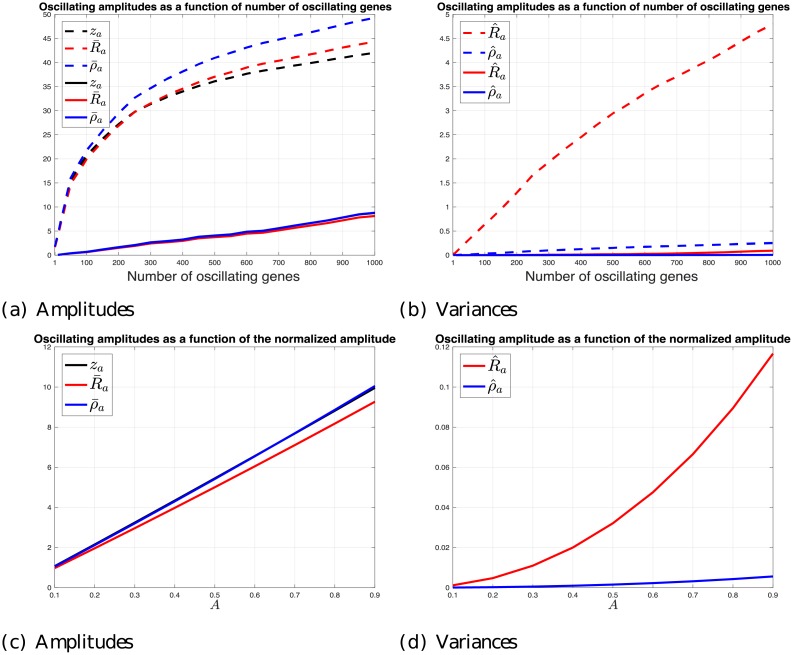
The effect of endogenous gene mRNA levels. (a) *z*_*a*_, ${\bar{R}}_{a}$, and ${\bar{\rho }}_{a}$ as a function of the number of oscillating genes chosen incrementally from a mRNA levels-sorted list of genes (see details in the Materials and methods section), (dashed-line), and genes chosen randomly from the gene list (solid-line), for *A* = 1/2. Note that the results for *z*_*a*_ and ${\bar{\rho }}_{a}$ when oscillating a typical gene set are very similar (the solid-line for *z*_*a*_ cannot be distinguished from the solid-line ${\bar{\rho }}_{a}$) (b) The corresponding variances. (c) *z*_*a*_, ${\bar{R}}_{a}$, and ${\bar{\rho }}_{a}$ as a function of the normalized amplitude *A* ∈ [0.1, 0.9] when oscillating the cell cycle related genes. (d) The corresponding variances.

At the first step, we aim at evaluating the impact of fluctuating mRNA levels of a *S. cerevisiae* endogenous gene set on the translation of the entire *S. cerevisiae* transcriptome. Fig 2 panels (a) and (b) depict the results as a function of the number of oscillating endogenous genes. In the figure we plot both the effect of the average/typical oscillating gene set, and the effect of the oscillating set with maximal mRNA levels (see the Materials and methods section for more details).

As can be seen, oscillating the mRNA levels of a typical large set of 1, 000 *S. cerevisiae* genes with normalized amplitude *A* = 1/2 is expected to typically induce an amplitude of about 9% on the rest of the genes translation rates; the maximal effect of a set of 1, 000 *S. cerevisiae* genes is very high and close to 50%. During the life cycle of a cell large sets of genes may fluctuate/oscillate together (e.g. due to a common regulatory mechanism) at the *transcription* level and the results reported here demonstrate that these oscillations should have non-negligible effect on the rest of the genes at the *translation* levels. Note that the results for *z*_*a*_ and ${\bar{\rho }}_{a}$ when oscillating a typical gene set are very similar (the solid-line for *z*_*a*_ cannot be distinguished from the solid-line for ${\bar{\rho }}_{a}$).

One phenomena that involves large scale gene expression oscillation is the cell cycle process. Ref. [17] identified 800 protein-encoding transcripts in *S. cerevisiae* that are cell cycle regulated, i.e. genes whose transcript levels vary periodically during the cell cycle process. These genes are involved in different cell cycle related functions such as cell cycle control, DNA replication, DNA repair, budding, glycosylation, nuclear division and mitosis. We evaluate the effect of oscillating these genes on the translation of the rest of the genes as a function of the normalized amplitude *A* ∈ [0.1, 0.9]. These are depicted in Fig 2 panels (c) and (d). It may be noticed that the amplitudes increase linearly with *A*, and that the amplitude of the free ribosomal pool and the translation amplitudes are very similar. Note that the variance of the steady-state mean density amplitude hardly change as a function of *A*, whereas the variance of the steady-state translation rate amplitude increases from zero to about 0.12 for *A* = 0.9. This suggests that the steady-state mean density amplitudes of all *S. cerevisiae* genes vary much less than the corresponding steady-state translation rate amplitudes. Since $\rho_{a}^{i}$ measures the average of the steady-state density amplitudes of gene *i*, it is indeed expected that its variance over all genes will be less than the variance of the steady-state translation rate amplitudes over all genes.

### The effect of heterologous gene mRNA levels and initiation rate on translation rate oscillations

Next, we aim at understanding the effect of oscillating the mRNA levels of a heterologous gene on the free ribosomal pool, and on the translation rate and ribosomal density of the endogenous genes. Note that there are many synthetic systems where the mRNA levels of a single heterologous gene occupy dozens of percentages of the total number of mRNAs in the cell (see, e.g., [49, 50]). This analysis should specifically provide some intuition related to the effect of synthetic gene expression oscillation circuits on the translation of the rest of the genes. (Note that there are many examples of synthetic genes with oscillatory mRNA levels [36, 51–57]). It should also teach us about the effect of fluctuations in the expression levels of highly expressed heterologous genes on the expression levels of the rest of the genes. To this end, we add to our whole-cell model a heterologous GFP gene with periodically varying mRNA levels.

**Fig 3:**
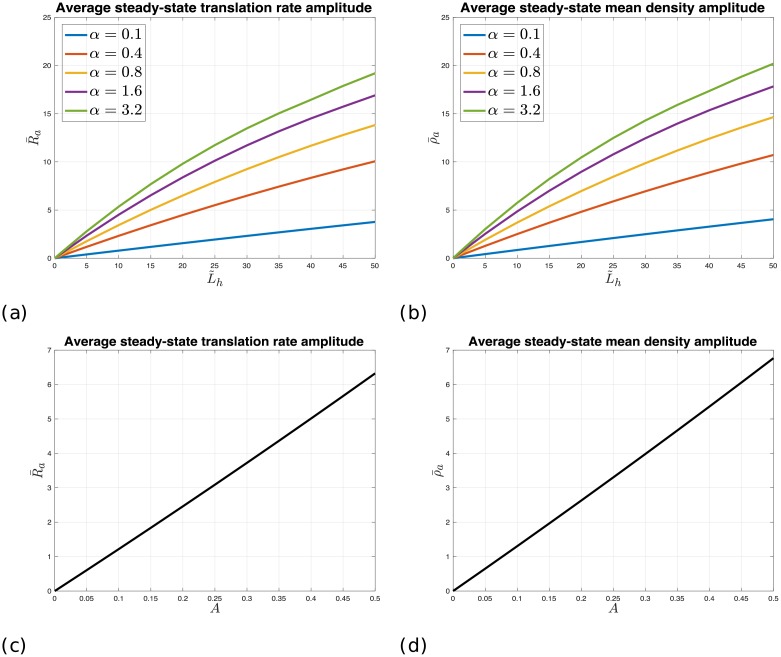
The effect of heterologous gene mRNA levels and initiation rates. (a) ${\bar{R}}_{a}$ as a function of ${\tilde{L}}_{h}$ and different values of *α*, for *A* = 1/2, *T* = 16, and $\bar{z}=30\%$. (b) ${\bar{\rho }}_{a}$ as a function of ${\tilde{L}}_{h}$ and different values of *α*, for *A* = 1/2, *T* = 16, and $\bar{z}=30\%$. (c) ${\bar{R}}_{a}$ as a function of *A* for *α* = 0.8, ${\tilde{L}}_{h}=20\%$, *T* = 16, and $\bar{z}=30\%$. (d) ${\bar{\rho }}_{a}$ as a function of *A* for *α* = 0.8, ${\tilde{L}}_{h}=20\%$, *T* = 16, and $\bar{z}=30\%$.

Fig 3 depicts the average steady-state translation rate amplitude (${\bar{R}}_{a}$) and mean density amplitude (${\bar{\rho }}_{a}$) for different (typical) values of GFP nominal mRNA levels ${\tilde{L}}_{h}$ and initiation rates *α* for *A* = 1/2, *T* = 16, and $\bar{z}=30\%$. It may be seen that both ${\bar{R}}_{a}$ and ${\bar{\rho }}_{a}$ increase with both *α* and ${\tilde{L}}_{h}$. This is expected since increasing *α* or ${\tilde{L}}_{h}$ increases the dynamic assignment of ribosomes to the GFP mRNAs, which in turn increases the impact of ribosomes assignment to the *S. cerevisiae* genes via the shared pool. For example, for ${\tilde{L}}_{h}=30\%$, ${\bar{R}}_{a}$ [${\bar{\rho }}_{a}$] ranges from about 2.5% [2.5%] to about 13.5% [14%]. Another observation is that the impacts on ${\bar{R}}_{a}$ and ${\bar{\rho }}_{a}$ are very similar. This suggests that by measuring the periodic amplitude of the translation rates at steady-state one can reasonably conclude the average amplitude of the mean ribosomal densities at steady-state.

Fig 3 also depicts ${\bar{R}}_{a}$ and ${\bar{\rho }}_{a}$ as a function of *A* ∈ (0, 1/2] for *α* = 0.8, ${\tilde{L}}_{h}=20\%$, *T* = 16, and $\bar{z}=30\%$. It may be noticed that the translation rate and ribosome density increase linearly with *A* ∈ (0, 1/2]. Similar observations were made for several other values of ${\tilde{L}}_{h}$ and *α*. We conclude that highly expressed heterologous genes can have an effect of up to about 20% on the amplitude of the translation rate and ribosome density of the rest of the endogenous genes. This should be considered when designing the properties of a synthetic circuit. Note that by the analysis done in the previous section, oscillations of large number of endogenous genes should also affect the heterologous genes.

### The effect of the codon compositions of a heterologous gene on the translation rate oscillations

In this section different synonymous substitutions are introduce to the heterologous GFP gene to study their effect, separately, on the translation of the endogenous genes. The goal here is to evaluate the effect of the coding region (and thus the induced ribosomal density and translation rate) on translation oscillation. In brief, we consider various variants of the GFP coding region; all of them code the same GFP protein but with different codons (a detailed description of each synonymously mutated GFP can be found in the Materials and methods section). The mutated GFP genes considered are:

1. GFP_HIGH_RD: designed for high ribosomal density (RD) and high “traffic jam”.
2. GFP_LOW_RD: designed for low ribosomal density and smooth “traffic flow”.
3. GFP_MDN_RD: designed for “median” ribosomal density.
4. GFP_SPD_TR: designed for high translation rate (TR) by utilizing synonymous codons with minimal decoding times.
5. GFP_SLW_TR: designed for low translation rate by utilizing synonymous codons with maximal decoding times.

Table 2 lists the steady-state translation rate *R* and mean densities *ρ* of each mutated GFP modeled to include initiation rate equals to 0.8 (which is the median initiation rate of the *S. cerevisiae* genome [47]). The table also lists two metrics (*η* and $\tilde{\eta }$) for ranking the codon decoding times of the coding region (named *decoding time measure (DTM)*). The DTM provides a score of how fast the ORF can be decoded; a value of zero means that it is composed of the fastest synonymous codons, and a larger value of DTM indicates that slower codons are used in the ORF. Specifically, in *η* all codons contribute equally to the DTM, whereas in $\tilde{\eta }$ the codon impact on the DTM increases as we move closer to the 3’-UTR end of the gene (see the Materials and methods section for more details).

**Table 2:** Mutated GFP genes translation properties.

Gene name	*ρ*	*R*	*η*	$\tilde{\eta }$
GFP (original)	0.4978	0.1667	0.0185	0.0372
GFP_HIGH_RD	0.6037	0.1498	0.0193	0.0458
GFP_LOW_RD	0.3734	0.1486	0.0206	0.0334
GFP_MDN_RD	0.3948	0.1489	0.0249	0.0432
GFP_SPD_TR	0.4935	0.1892	0	0
GFP_SLW_TR	0.4812	0.1456	0.0399	0.0792

The following may be concluded from Table 2:

- The maximal [minimal] steady-state mean density is in the case of GFP_HIGH_RD [GFP_LOW_RD]. This is expected since GFP_HIGH_RD [GFP_LOW_RD] was designed to create “traffic jam” [“traffic flow”] of ribosomes at steady-state. Specifically, GFP_HIGH_RD uses 62% more ribosomal density at steady-state compared to the steady-state density in GFP_LOW_RD. This implies that on average about 60% [37%] of the codons in GFP_HIGH_RD [GFP_LOW_RD] are covered by ribosomes.
- The maximal [minimal] steady-state translation rate is in the case of GFP_SPD_TR [GFP_SLW_TR]. This is expected since GFP_SPD_TR [GFP_SLW_TR] consists of codons with minimal [maximal] decoding times. This implies that the maximal [minimal] steady-state translation rate of the GFP protein (over all possible synonymous substitutions) is 0.1892 [0.1456] (i.e. we can increase the translation rate of the GFP gene by at most 30% using synonymous substitutions).
- The steady-state translation rates of the mutated genes GFP_LOW_RD and GFP_HIGH_RD are similar, although GFP_LOW_RD uses 38% less ribosomal density at steady-state.
- *η* correlates well with the steady-state translation rate.

**Fig 4:**
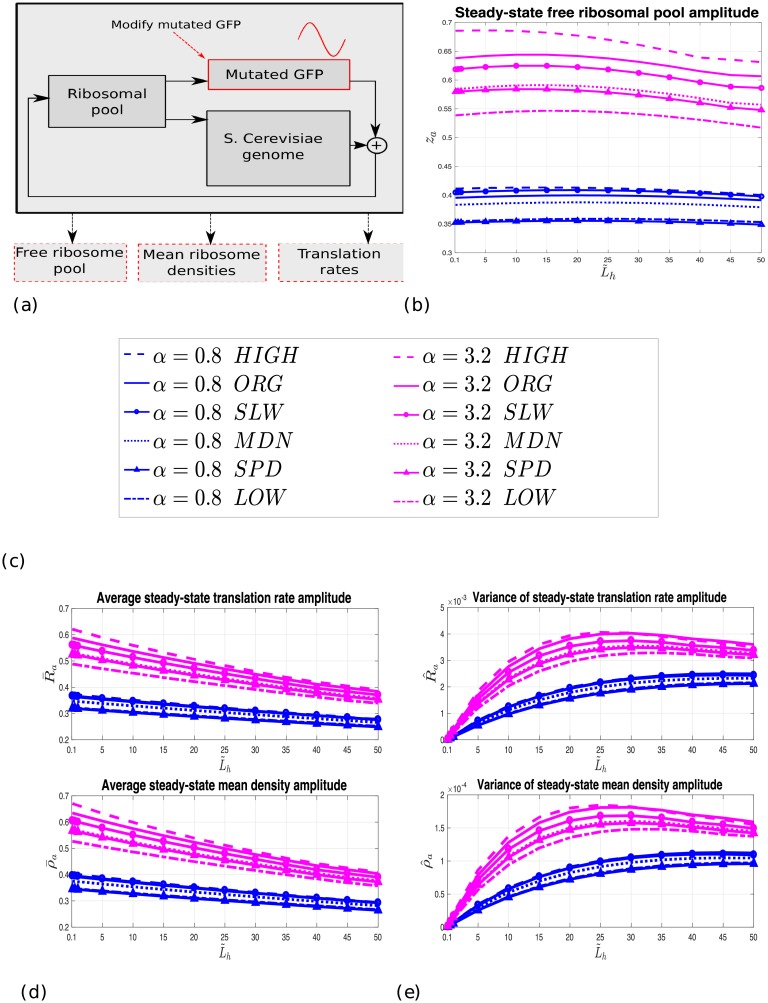
The effect of heterologous gene mRNA levels and elongation rates. (a) Block diagram of the current test. (b) Normalized *z*_*a*_ as a function of ${\tilde{L}}_{h}$, *α* = 0.8 and *α* = 3.2 for *A* = 1/2, *T* = 16, and $\bar{z}=30\%$. “ORG” denotes the original (non-mutated) GFP. (c) Legend of the sub-figures. The up-to-down order corresponds to the performance ranking, per ${\tilde{L}}_{h}$ value, in sub-figures (b), (d), and (e), i.e. HIGH results in the largest measurement values, followed by ORG, etc. (d) Normalized ${\bar{R}}_{a}$ (upper figure) and normalized ${\bar{\rho }}_{a}$ (lower figure) as a function of ${\tilde{L}}_{h}$, *α* = 0.8 and *α* = 3.2 for *A* = 1/2, *T* = 16, and $\bar{z}=30\%$. (e) Normalized ${\hat{R}}_{a}$ (upper figure) and normalized ${\hat{\rho }}_{a}$ (lower figure) as a function of ${\tilde{L}}_{h}$, *α* = 0.8 and *α* = 3.2 for *A* = 1/2, *T* = 16, and $\bar{z}=30\%$.

Fig 4 depicts the translation *normalized* statistics as a function of the nominal mRNA levels ${\tilde{L}}_{h}$, for *α* = 0.8 and *α* = 3.2, for each of the GFP mutated genes, when translated (separately) with the *S. cerevisiae* gene pool, for *A* = 1/2, *T* = 16, and $\bar{z}=30\%$. Each data point in the figure represents the corresponding statistics per 600 mutated GFP mRNAs (1% of the total *S. cerevisiae* mRNA levels), i.e. we divide the statistics values by the corresponding ${\tilde{L}}_{h}$ and multiply by 600. This represents the impact on the translation process per a unit of 600 GFP mRNAs (a normalized measure can then be used to compare results between different values of ${\tilde{L}}_{h}$).

We first observe that the normalized statistics increase with *α* for each ${\tilde{L}}_{h}$ value. This is obviously expected since large values of *α* imply high periodic variations of assigned ribosomes to the GFP mRNAs, and thus also to the *S. cerevisiae* genes mRNAs (due to the shared pool), and so we expect the amplitudes of the free pool, translation rates and mean densities to increase. We also note that the statistics variations over the different mutations increase with *α*. For example, for ${\tilde{L}}_{h}=25\%$, the normalized *z*_*a*_ varies between 0.55% and 0.67% (in case of *α* = 3.2), and between 0.35% and about 0.4% (in the case of *α* = 0.8). This is expected since, for example, a low value of *α* means that the initiation is the rate limiting factor, and in this case the GFP ORF mutations (affecting the elongation rates) less affect the parameters.

In addition, it may be seen that the normalized statistics maintain a particular ranking for different ${\tilde{L}}_{h}$ values: they achieve their maximal values when oscillating the GFP_HIGH_RD mutation, are reduced when oscillating the GFP original gene, and achieve their minimal values when oscillating the GFP_LOW_RD mutation. For example, for ${\tilde{L}}_{h}=10\%$ and *α* = 3.2, the normalized ${\bar{R}}_{a}$ is about 0.57% when oscillating GFP_HIGH_RD, is about 0.55% when oscillating GFP, and is about 0.45% when oscillating GFP_LOW_RD. This correlates with the mean steady-state ribosomal densities of these mutations, as well as with their non-homogeneous DTMs ($\tilde{\eta }$). This suggests that mRNAs with “traffic jams” at steady-state (i.e. mRNAs that occupy large number of ribosomes at steady-state) have a substantial impact on the translation of the other genes via the shared ribosomal pool.

The normalized *z*_*a*_, ${\bar{R}}_{a}$ and ${\bar{\rho }}_{a}$ seem to slightly decrease with ${\tilde{L}}_{h}$, implying that the non-normalized parameters increase sub-linearly with ${\tilde{L}}_{h}$. Oscillating the mRNA levels of the GFP gene increases and decreases periodically the assigned number of ribosomes to the GFP mRNAs, which in turn decreases and increases periodically the amount of free ribosomes, respectively. This affects the actual initiation rate to the mRNAs. However, due to the finite, shared pool of ribosomes, the oscillation effect caused by an increase in mRNA levels admits a linear region which is eventually saturated (similar to most physical systems). Finally, it may be observed that the corresponding variances increase with both ${\tilde{L}}_{h}$ and *α*, indicating, as expected, that for large oscillating mRNA levels, and/or initiation rates, the variations of the amplitudes over all genes increase. Note that the variance values are few order of magnitudes less than the corresponding average values; for example, for ${\tilde{L}}_{h}=20\%$ and *α* = 3.2, ${\hat{R}}_{a}$ [${\hat{\rho }}_{a}$] is about 0.7% [0.03%] of the corresponding average values.

In general, both the steady-state translation rate and the mean density of the mutated or the original GFP gene affect the parameters. For example, the effect of the gene GFP_SPD_TR on the statistics is less severe than the effect of the gene GFP_MDN_RD, even-though GFP_SPD_TR consumes more (by 25%) ribosomes at steady-state (see Table 2). However, the steady-state translation rate of GFP_SPD_TR is larger (by about 26%) than the steady-state translation rate of GFP_MDN_RD, thus ribosomes in the GFP_SPD_TR mutation case are released faster to the pool and thus are available more for translating other genes.

In addition, we can observe a ‘diminishing marginal utility’ effect: the results depicted in Fig 4 suggest that oscillating a larger number of mRNAs of the mutated GFP gene decreases the amplitude of the free pool and of the genes translation rate and mean density *per GFP mRNA level*. This effect is partially due to the limited and shared ribosome pool.

**Fig 5:**
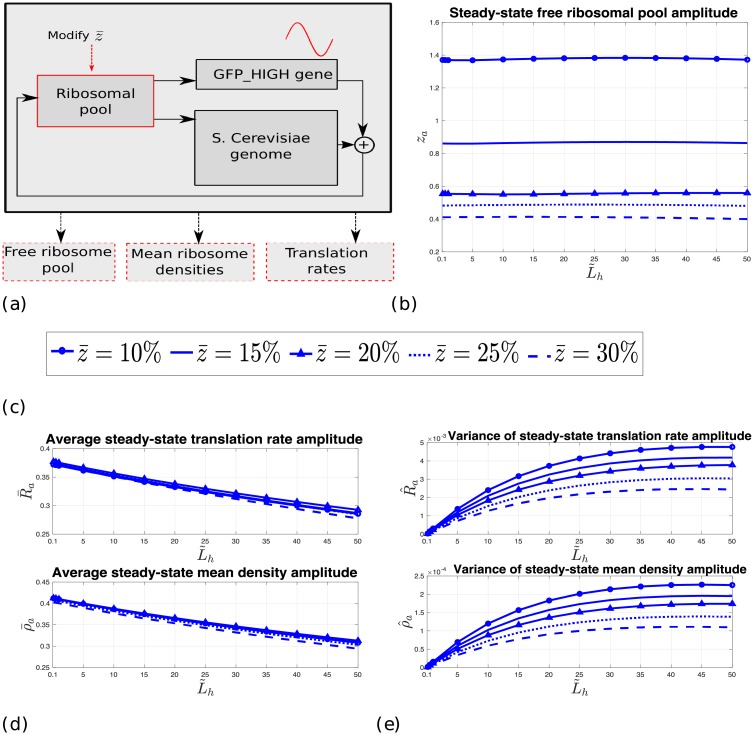
The effect of the average ribosomal pool. (a) Block diagram of the current test. (b) Normalized *z*_*a*_ as a function of ${\tilde{L}}_{h}$ of GFP_HIGH_RD, for *α* = 0.8, *A* = 1/2, *T* = 16, and different values of $\bar{z}$. (c) Legend of the sub-figures. The right-to-left order corresponds to the performance ranking, per ${\tilde{L}}_{h}$ value, in sub-figures (b), (d), and (e), i.e. $\bar{z}=10\%$ results in the largest measurement values, followed by $\bar{z}=15\%$, etc. (d) Normalized ${\bar{R}}_{a}$ (upper figure) and normalized ${\bar{\rho }}_{a}$ (lower figure) as a function of ${\tilde{L}}_{h}$ of GFP_HIGH_RD, for *α* = 0.8, *A* = 1/2, *T* = 16, and different values of $\bar{z}$. (e) Normalized ${\hat{R}}_{a}$ (upper figure) and normalized ${\hat{\rho }}_{a}$ (lower figure) as a function of ${\tilde{L}}_{h}$ of GFP_HIGH_RD, for *α* = 0.8, *A* = 1/2, *T* = 16, and different values of $\bar{z}$.

Fig 5 depicts the translation *normalized* statistics when oscillating the mutated GFP_HIGH_RD gene for several values of the average steady-state free ribosomal pool $\bar{z}$. It may be seen that *z*_*a*_ decreases with $\bar{z}$, whereas ${\bar{R}}_{a}$ and ${\bar{\rho }}_{a}$ are slightly affected by $\bar{z}$. For example, for ${\tilde{L}}_{h}=30\%$ and *α* = 0.8, the normalized *z*_*a*_ decreases from about 1.4% for $\bar{z}=10\%$ to about 0.4% for $\bar{z}=30\%$, whereas both the normalized ${\bar{R}}_{a}$ and ${\bar{\rho }}_{a}$ hardly vary and are equal to about 0.32% and 0.34%, respectively. One possible explanation for this is as follows: As $\bar{z}$ decreases (i.e. as less ribosomes are free thus more are assigned to the mRNAs) the effective initiation rates to the mRNA increases. This increases the oscillation amplitude induced by the GFP_HIGH_RD mRNAs, and thus the relative effect on $\bar{z}$ increases (recall that *z*_*a*_ denotes the free pool oscillation amplitude relative to $\bar{z}$). On the other hand, an increase in the effective initiation rates increases both the steady-state translation rates and mean densities of all the *S. cerevisiae* mRNAs, and so the effect on ${\bar{R}}_{a}$ and ${\bar{\rho }}_{a}$ is small. However, as suggested by Fig 5, the corresponding variances increase slightly as $\bar{z}$ decreases, implying that the amplitude variations over all genes do not change much as $\bar{z}$ decreases from 30% to 10%. Note that, again, the variance values are few order of magnitudes less than the corresponding average values (for example, for ${\tilde{L}}_{h}=20\%$, ${\hat{R}}_{a}$ [${\hat{\rho }}_{a}$] is about 1.0% [0.05%] of the corresponding average values).

The results depicted in Fig 5 suggest that the fluctuations of the translation rates and mean ribosomal densities are hardly affected by the affinity of ribosomes to the mRNA molecules (this affinity may be controlled by initiation efficiency, for example). However, the fluctuations of the free ribosomal pool increase as more ribosomes are translating the mRNA molecules.

As another example, Table 3 depicts the (non-normalized) statistics when oscillating the mutated GFP_HIGH_RD gene for *A* = 0.35, *T* = 16, *α* = 0.8, ${\tilde{L}}_{h}=20\%$, and for several values of the average free ribosomal pool at steady-state $\bar{z}$. The same conclusions can be derived here as well.

**Table 3:** Statistics as a function of the average free pool at steady-state for *A* = 0.35, *T* = 16, *α* = 0.8, and ${\tilde{L}}_{h}=20\%$.

$\bar{z}$	*z*_*a*_	${\bar{R}}_{a}$	${\bar{\rho }}_{a}$
30%	5.4706%	4.3655%	4.6672%
25%	6.4515%	4.4146%	4.7452%
20%	7.3070%	4.4660%	4.8269%
15%	11.4549%	4.4055%	4.7880%
10%	18.1985%	4.3827%	4.7901%

In summary, the current subsection teaches us that when designing highly expressed heterologous genes that are expected to fluctuate/oscillate we should carefully choose their codons composition: to induce low effect on the other genes we should minimize the ribosome density, and on the other hand high ribosomal density results in large effect on the other genes. As was demonstrated here the exact profiles that maximize/minimize ribosome densities are not simply the ones with optimal/slowest codons along the coding region, respectively; thus, it is important to develop models and algorithms for engineering and manipulating ribosome density of endogenous and heterologous genes.

The results reported here with the heterologous gene may be further validated experimentally in the future using in-vitro and/or in-vivo systems with oscillating GFP proteins [51]. However, we believe that with the current experimental approach it should be challenging to directly study the coupling we reported here due to the following reasons. First, oscillating endogamous systems probably includes various effects and feedbacks that may “cancel” or blur the phenomena presented here. Second, in order to be able to measure, with the current techniques, the effect reported here large portion of the mRNA molecules in the cell should oscillate. Finally, this study analyzes oscillations during the translation stage. Thus, to study them one should directly measure translation rate; the conventional experimental approaches (e.g. RNA-seq, ribo-seq or approaches based on quantitative mass spectrometry) measure variables that are expected to be related/correlated with the translation rate but are not the actual translation rate.

Our results should be specifically considered when designing large intra-cellular circuits with many components/genes. In such cases, among others, the oscillation in transcription levels of some parts of the circuit should affect the other part of the circuit. We provide here some initial guidelines related to this topic. First, if we are not interested in cross-talk between the different oscillating genes we should engineer their transcript to minimize the induced oscillations (e.g. designing codon profiles that minimize ribosome density and if possible decrease their initiation rate). Second, in some cases we may want to design genes that induce oscillations on the rest/other genes; in these cases, we will design them accordingly (e.g. high initiation rate and ribosome density). Third, our (or similar) models can be used to estimate potential “noise” due to oscillation cross-talk. These estimations can be considered when designing the circuit and assuring its performance.

The goal of this study is to understand and carefully quantify the impact of oscillations over wide range of conditions and parameters (e.g., large range of *A*, ${\tilde{L}}_{h}$, *α*, $\bar{z}$, and different GFP mutations). This is important, as the severity of the oscillations impact (in terms of its phenotypic or biological-significant effect) is, in general, gene and condition specific. It might depend on the function of the genes (e.g. structural genes, transcription factors, signaling proteins, etc.), the exact condition (e.g. initiation rate, mRNA levels), the organisms type, etc.

The computational model used in this study is deterministic, enabling rigorous analysis of its properties using tools from systems and control theory. In addition, it was shown to admit high correlation with the stochastic TASEP model of translation (e.g. see [45]), and furthermore using it to simulate large-scale translation with competition is simple. The processes in the cell are stochastic in nature, and future study may employ stochastic whole cell models to study the effect of oscillations in a “noisy” environment. In S6 Fig we provide initial results that suggest that noise in the model parameters should not affect our conclusions.

It is important to emphasize that the results reported here are relevant also in cases where the time scales of translation and cell cycle differ. Note that it has been suggested that translation of cell cycle related genes is regulated by periodically varying tRNA levels [18]. This implies, among others, that the time scales are quite similar. Specifically, the translation time in general can be longer than the cell cycle period. For concreteness, consider the case of *S. cerevisiae*. The cell cycle period in *S. cerevisiae* is less than 87 minutes [58]. Cell cycle period can be much shorter in eukaryotes; for example, it was reported that the duration of cell cycle in early embryo of the fruit fly D. melanogaster is only eight minutes [59]. The translation rate in *S. cerevisiae* was estimated to be higher than 0.956 codons per second (the slowest codon is CUU) [60] with average rate over all codons of 10 codons per second (in mouse the average codon translation rate was estimated to be about five codons per second [61]). In practice, this rate can be much slower due to strong folding of the mRNA molecule and interaction of the translated amino-acid peptide with the exit channel of the ribosome [62, 63]. In *S. cerevisiae* the ORF length is between 75 and 14, 733 nucleotides. The longest gene corresponds to an upper bound on the translation time of a gene, which is about 82 minutes (assuming a lower bound on translation rate of one codon per second, which may be lower in practice), an estimated translated time of this protein based on mean codon translation time is 8.2 minutes. In mammals the mean codon decoding time is five codons per second, and the longest human protein (Titin–TTN), which consists of 33, 000 amino acids, corresponds to estimated translation time of 110 minutes. This suggests that periodically varying mRNA levels in cell cycle related genes may be similar to the time scale of mRNA translation.

We will conclude with the main lessons from the analysis performed here based on our whole-cell computational model: 1) Competitions for limited resources in the cell lead to indirect couplings between the gene expression stages, and these couplings must be considered when analyzing the cellular economy of the cell; 2) A whole-cell computational model of translation that takes into account fundamental properties of translation, with parameters estimated based on experimental measurements, can comprehensively quantify the effect of oscillations on the ribosomal densities and translation rates of all genes; 3) Careful considerations must take place when designing highly express heterologous genes that are expected to fluctuate, as their codon compositions and translation initiation rates may have high effect on all genes translation rate. We demonstrate specific cases with high and low effect on fluctuations; 4) Quantitative estimation (based on parameters estimated from experimental data) of the magnitude of these oscillations in endogenous and heterologous genes is provided here; and 5) The conclusions reported here in general should also be relevant to other aspects of gene expression and/or intracellular phenomenon. For example, when considering oscillations in tRNA levels, the number of DNA copies of a virus, intracellular transport factors, etc.

## Materials and methods

### Fluctuating mRNA levels in endogenous genes

We first sort the *S. cerevisiae* genes according to mRNA levels and evaluate the steady-state mean density and translation rate amplitudes as a function of the number of oscillating genes chosen sequentially from the sorted list of genes, starting from the gene with the largest mRNA levels (dashed-lines in Fig 2, panels (a) and (b)). For example, when using *p* genes with oscillating mRNA levels, the *p* genes with the largest mRNA levels are used. This provides a bound on the maximal oscillating amplitudes when any *arbitrary* *p* *S. cerevisiae* genes are oscillating.

The “typical” selected genes were chosen *randomly* from the *S. cerevisiae* gene pool. Here the oscillation amplitudes and variances for each number of “typical” oscillating genes set is averaged over 30 repetitions. The results of oscillating these genes are depicted, using solid-lines, in Fig 2, panels (a) and (b).

The assumption in this study is that large set of genes can be regulated (oscillate) independently of the ribosomal pool. This is motivated by: 1) The regulations at the translation and transcription stages are not tightly coupled [64]; 2) There may be delays between the two stages [65]; 3) In the case of heterologous genes (and the corresponding promoters and gene expression circuits) that are engineered by design there is no reason to assume that the ribosome pool is also regulated.

### Cell cycle genes

Ref. [17] identified 800 protein-encoding transcripts in *S. cerevisiae* that are cell cycle regulated. We evaluate the parameters when oscillating 770 of these genes, as we lack mRNA measurements for 30 of the reported 800 cell cycle related genes. The 770 cell cycle genes used are listed in S1 Table. Table 4 lists the 30 genes we lack mRNA measurements for.

**Table 4:** The 30 (out of 800) cell cycle related genes reported in [17] that we lack mRNA measurements for (and thus are not used in our simulations).

YJL195C	YDL163W	YJL018W	YJL119C	YCL012W	YCRX05W
YJL067W	YCL060C	YIL025C	YLR302C	YNL171C	YGR259C
YLR013W	YLR458W	YDL096C	YCL062W	YPR014C	YIL168W
YKL177W	YLR235C	YPR076W	YCLX09W	YML033W	YFL006W
YGR219W	YLR236C	YDR355C	YCL013W	YML035C-A	YIL167W

### Whole-cell computational model overview

The *ribosome flow model network with pool (RFMNP)* [43] is a deterministic computational model for large-scale simultaneous mRNA translation and competition for ribosomes. It is based on combining several *ribosome flow models with input and outputs (RFMIOs)* [45, 66], interconnected via a pool of free ribosomes. Each gene is modeled by a single RFMIO, and all the RFMIOs are sharing the same pool of ribosomes. The dynamics of the system is expressed by a set of ordinary differential equations that describes the time evolution of the ribosomal densities in the different RFMIOs and the free pool. In this paper we utilize the RFMNP to simulate a whole-cell *S. cerevisiae* simultaneous translation and competition for ribosomes. Each of the *S. cerevisiae* gene (and the GFP gene) is modeled by a single RFMIO. We next describe in details the RFMIO and the RFMNP.

### The ribosome flow model with input and output (RFMIO)

**Fig 6:**
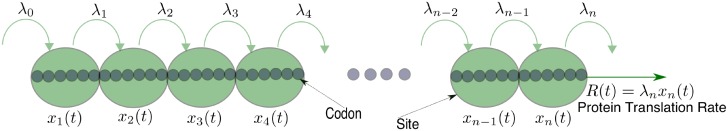
The RFM as a chain of *n* sites of codons. Each site is described by a state variable *x*_*i*_(*t*) ∈ [0, 1], expressing the normalized ribosome occupancy at site *i* at time *t*. λ_0_ is the initiation rate, and λ_*i*_ is the elongation rate from site *i* to site *i* + 1. Translation rate at time *t* is *R*(*t*) ≔ λ_*n*_*x*_*n*_(*t*).

The *ribosome flow model* (RFM) [45] is a *deterministic* mathematical model for mRNA translation that can be derived by a mean-field approximation of an important model from statistical physics called the *totally asymmetric simple exclusion process (TASEP)* (see, e.g., [67] and [68]). In the RFM, mRNA molecules are coarse-grained into *n* consecutive sites of codons. The state variable $x_{i}(t):{\mathbb{R}}_{+}\to [0,1]$, *i* = 1, …, *n*, describes the normalized ribosomal occupancy level at site *i* at time *t*, where *x*_*i*_(*t*) = 1 [*x*_*i*_(*t*) = 0] indicates that site *i* is completely full [empty] at time *t*. The model includes *n* + 1 positive parameters that regulate the transition rate between the sites: the initiation rate into the chain λ_0_, the elongation (or transition) rate from site *i* to site (*i* + 1) λ_*i*_, *i* = 1, …, *n* − 1, and the exit rate λ_*n*_ (see Fig 6).

The dynamics of the RFM with *n* sites is given by *n* nonlinear first-order ordinary differential equations:

$$\begin{array}{cc} {\dot{x}}_{1} & =\lambda_{0}\left(1-x_{1}\right)-\lambda_{1}x_{1}\left(1-x_{2}\right),\\ {\dot{x}}_{2} & =\lambda_{1}x_{1}\left(1-x_{2}\right)-\lambda_{2}x_{2}\left(1-x_{3}\right),\\ {\dot{x}}_{3} & =\lambda_{2}x_{2}\left(1-x_{3}\right)-\lambda_{3}x_{3}\left(1-x_{4}\right),\\ & \vdots \\ {\dot{x}}_{n-1} & =\lambda_{n-2}x_{n-2}\left(1-x_{n-1}\right)-\lambda_{n-1}x_{n-1}\left(1-x_{n}\right),\\ {\dot{x}}_{n} & =\lambda_{n-1}x_{n-1}\left(1-x_{n}\right)-\lambda_{n}x_{n}. \end{array}$$ (2)

If we let *x*_0_(*t*) ≔ 1 and *x*_*n*+1_(*t*) ≔ 0, then (2) can be written more succinctly as

$$\begin{array}{c} {\dot{x}}_{i}=h_{i-1}\left(x\right)-h_{i}\left(x\right),\;i=1,\&,n, \end{array}$$ (3)

where *h*_*i*_(*x*) ≔ λ_*i*_*x*_*i*_(1 − *x*_*i*+1_). This can be explained as follows. The flow of ribosomes from site *i* to site *i* + 1 at time *t* is λ_*i*_*x*_*i*_(1 − *x*_*i*+1_). This flow increases with the density at site *i*, and decreases as site *i* + 1 becomes fuller. This corresponds to a “soft” version of a simple exclusion principle. Since the ribosomes have volume, the input rate to site *i* decreases as the number of ribosomes in that site increases. Note that the maximal possible flow from site *i* to site *i* + 1 is the transition rate λ_*i*_. Thus, Eq (3) simply states that the change in the density at site *i* at time *t* is the input rate to site *i* (from site *i* − 1) minus the output rate (to site *i* + 1) at time *t*.

The ribosome exit rate from site *n* at time *t* is equal to the protein translation rate at time *t*, and is denoted by *R*(*t*) ≔ λ_*n*_ *x*_*n*_(*t*).

Denote by *x*(*t*, *a*) the solution of (3) at time *t* ≥ 0 for the initial condition *x*(0) = *a*. Since the state-variables correspond to normalized occupancy levels, we always assume that *a* belongs to the closed *n*-dimensional unit cube $\begin{array}{c} C^{n}≔\left\{x\in R^{n}:x_{i}\in \left[0,1\right],i=1,\&,n\right\}. \end{array}$ Let int(*C^n^*) denote the interior of *C*^*n*^. Ref. [69] showed that the RFM is a *tridiagonal cooperative dynamical system* [70], and that this implies that (2) admits a *unique* steady-state point *e* = *e*(λ_0_,…λ_n_) ∈ int(*C^n^*), that is globally asymptotically stable, that is, lim_*t*→∞_ *x*(*t*, *a*) = *e*, for all *a* ∈ *C*^*n*^ (see also [71]). In particular, the translation rate converges to the steady-state value *R* ≔ λ_*n*_*e*_*n*_. We denote by

$$\begin{array}{c} \rho ≔\frac{\sum_{i=1}^{n}e_{i}}{n} \end{array}$$

the steady-state mean ribosomal density along the mRNA.

The RFM can be extended into a single-input single-output (SISO) control system, by defining the translation rate as the output, and by introducing a time-varying input control $u:{\mathbb{R}}_{+}\to {\mathbb{R}}_{+}$ representing the flow of ribosomes from the “outside environment” into the mRNA (which is related to the rate ribosomes diffuse to the 5’end (in eukaryotes) or the RBS (in prokaryotes) of the mRNA). This is referred to as the *RFM with input and output (RFMIO)* [66]. Thus, the equation for the change in the density at site 1 in the RFMIO becomes

$${\dot{x}}_{1}=\lambda_{0}u(1-x_{1})-\lambda_{1}x_{1}(1-x_{2}),$$

and all other equations for ${\dot{x}}_{i}$, *i* = 2, …, *n*, are the same as in the RFM. The RFMIO can then be written in a compact-form as

$$\begin{array}{ll} \dot{x} & =f(x,u),\\ y & =\lambda_{n}x_{n}, \end{array}$$ (4)

where *y* denotes the output.

In this study, each *S. cerevisiae* gene is modeled by a RFMIO, where each RFMIO site contains 10 consecutive codons (the ribosome footprint is assumed to be about 10 codons wide).

### RFM network with a pool (RFMNP)

In [43], a network of *m* RFMIOs interconnected via a pool of free ribosomes (called the *RFM network with pool (RFMNP)*) was introduced for analyzing large-scale translation while competing for the available, limited ribosomal resource. Competition for the available ribosomal resource leads to indirect coupling between the different mRNAs. For example, if more ribosomes bind to a certain mRNA molecule then the pool of free ribosomes in the cell is depleted, and this may lead to lower initiation rates in the other mRNAs.

Let $z(t):{\mathbb{R}}_{+}\to {\mathbb{R}}_{+}$ denote the free ribosomal pool occupancy at time *t*. For an RFMNP with *m* RFMIOs, let *n*^*j*^, *i* = *j*, …, *m*, denote the *j*th RFMIO dimension, *y*^*j*^ ≔ *R*^*j*^(*t*) its output rate at time *t*, and $\lambda_{0}^{j},\ldots ,\lambda_{n^{j}}^{j}$ its transition rates. The input to the *j*th RFMIO is *u^j^* = *G^j^*(*z*) where the function $G^{j}(\cdot ):{\mathbb{R}}_{+}\to {\mathbb{R}}_{+}$ satisfies: (1) *G*^*j*^(0) = 0; (2) *G*^*j*^(*z*) is strictly increasing on ${\mathbb{R}}_{+}$; and (3) for all *z* > 0 sufficiently small *G*^*j*^(*z*) is linearly proportional to *z*. Typical examples are *G*^*j*^(*z*) = *z*, and *G*^*j*^(*z*) = *a*_*j*_ tanh(*z*/*b*_*j*_) with *a*_*j*_, *b*_*j*_ > 0 (see S1 Fig and [43] for more details). Thus, the RFMNP is given by

$$\begin{array}{cc} {\dot{x}}^{1} & =f\left(x^{1},u^{1}\right),y^{1}=\lambda_{n^{1}}^{1}x_{n^{1}}^{1},\\ \vdots & \\ {\dot{x}}^{m} & =f\left(x^{m},u^{m}\right),y^{m}=\lambda_{n^{m}}^{m}x_{n^{m}}^{m}, \end{array}$$ (5)

and

$$\begin{array}{c} \dot{z}=\sum_{j=1}^{m}y^{j}-\sum_{j=1}^{m}\lambda_{0}^{j}\left(1-x_{1}^{j}\right)G^{j}\left(z\right). \end{array}$$ (6)

Eq (6) implies that the change in the free pool, as a function of time, is the sum of all output rates of the RFMIOs (input flow to the free pool) minus the total flow of ribosomes that bind to the mRNA molecules (output flow from the free pool). The RFMNP is then a dynamical system with $\left(1+\sum_{j=1}^{m}n^{j}\right)$ state-variables. Since the RFMNP is a closed system, the total number of ribosomes $H(t)≔z(t)+\sum_{j=1}^{m}\sum_{i=1}^{n^{j}}x_{i}^{j}(t)$ is conserved, that is *H*(*t*) ≡ *H*(0) for all *t* ≥ 0.

It was proven in [43] that for any given number of total ribosomal pool *H*(0), the RFMNP admits a unique steady-state point that depends on the rates and *H*(0) but not on the initial conditions. Furthermore, if one or more of the RFMIOs rates are time-periodic functions, with a common minimal period *T* > 0, then the RFMNP entrains to the periodic excitations in the $\lambda_{i}^{j}\text{s}$, i.e. every state-variable converges to a periodic solution with period *T*. This also means that each of the translation rates and mean densities converge to periodic solutions with period *T*. Thus, we do not need to evaluate different values of *T*, and the value *T* = 16 is used throughout this paper (e.g. using *T* = 20 instead yields the same behavior but with periodicity *T* = 20).

In this paper we simulate the RFMNP while periodically changing the mRNA levels of the heterologous GFP gene or several endogenous genes. Assume, for example, that the GFP mRNA levels are changing (periodically) between a minimal value of *β*_1_ > 0 and a maximal value of *β*_2_ > *β*_1_. It is straightforward to verify that this is equivalent to an RFMNP with *β*_2_ copies of the gene GFP, while periodically changing the initiation rates of these copies. Thus, [43] provides a rigorous proof to the periodicity we observed at steady-state in the state-variables. Finally, the parameters of the model used here are based on [72]; see more details below.

### Codons decoding times

We use ribo-seq data to infer the codon decoding rates [73], and normalize these rates so that the median codon elongation rate of all *S. cerevisiae* mRNAs becomes 6.4 codons per second [48]. This holds for *all* endogenous genes and the GFP. The ribo-seq data and the decoding rates are used also for inferring the initiation rates. The ribo-seq data and mRNA levels were taken from [74]; the number of *S. cerevisiae* ribosomes used in the simulation is 200,000 [75], with 60,000 mRNAs [46], scaled according to the mRNA levels from [74]. Thus, the correlations between the predicted ribosome densities from our model and measured ribosome densities in the analyzed conditions are very high (correlation coefficient *r* > 0.7 for sites size of 10 codons) and is similar to the correlations between two experimental replications in the field [76]. Note that the large-scale measurements of mRNA levels and ribosome profiling suggest that almost all genes have certain mRNA levels and ribosome densities; this suggests that most of the genes are transcribed/translated at the same time but at (possibly extremely) different rates/levels (the differences among genes can be very significant: up to four orders of magnitudes).

### Modeling a single *S. cerevisiae* mRNA translation by the RFMIO

Let *q* ≔ 10 denote the number of codons per RFMIO site. Given an *S. cerevisiae* gene ORF consisting of *K* codons (excluding the stop codon), we model it using RFMIO with *n* sites as follows. The mRNA is divided into (*n* + 1) pieces: the first piece contains (*q* − 1) codons (that are also related to later stages of initiation [14]), pieces 2 to *n* contain each *q* non-overlapping codons, and the last piece contains between *q*/2 and 3*q*/2 codons. For example, for *q* = 10 and *K* = 146, the first piece contains 9 codons, pieces 2 to 14 contain each 10 codons, and piece 15 contains 7 codons, thus *n* = 14. The first piece corresponds to λ_0_, and pieces 2 to *n* + 1 correspond to λ_1_ to λ_*n*_, respectively, as described next.

The initiation rate (that corresponds to the first piece) is estimated based on the ribosome density per mRNA level, as this value is expected to be approximately proportional to the initiation rate when initiation is the rate limiting factor [45, 77]. We apply a normalization that sets the median initiation rate of all *S. cerevisiae* mRNAs to 0.8 [47].

The RFMIO rates, per *S. cerevisiae* gene, are then set as follows:

- For all *i* ∈ {1, …, *n*}, $\lambda_{i}≔1/(\sum_{k=1}^{L}\tau_{k})$, where *L* is the number of codons in piece (*i* + 1), and *τ*_*k*_ is the decoding time of the *k*’th codon in that piece (see S2 Table). Indeed *L* = *q* for *i* = 1, …, *n* − 1, and *q*/2 ≤ *L* ≤ 3*q*/2 for *i* = *n*.
- $\lambda_{0}≔1/(p^{-1}+\sum_{k=1}^{q-1}\tau_{k})$, where *p* is the estimated initiation rate of the corresponding mRNA, and *τ*_*k*_ is the decoding time of the *k*’th codon in the first piece.

### Decoding-time measure (DTM)

Let *τ*_*i*_ denote the decoding time of codon *i* in the ORF, and let *ψ*(*i*) denote the minimum among the decoding times of codon *i* and its synonymous mutations. Define the *decoding-time measure (DTM)* of a gene by

$$\begin{array}{c} \eta ≔\frac{\sum_{i=1}^{K}\left(\tau_{i}-\psi \left(i\right)\right)w_{i}}{K}, \end{array}$$ (7)

where *K* denotes the number of codons in the ORF (excluding the stop codon), and *w*_*i*_ > 0, *i* = 1, …, *K*, is the weight given to the non-negative cost (*τ*_*i*_ − *ψ*(*i*)). The DTM then provides a score of how fast the ORF can be decoded; a value of zero means that the ORF is composed from the fastest synonymous codons, and a larger value of *η* indicates that slower codons are used in the ORF. One might expect that in general *η* should be inversely proportional to the steady-state translation rate. However, since *η* doesn’t provide information about the distribution of the decoding time costs along the ORF, this might not always hold. For example, a slow codon in the middle of the ORF can impact the steady-state translation rate more than a slow codon in the boundaries. Another possible interpretation of *η* is in describing the “speed-budget” relative to the optimum (*η* = 0 corresponds to the fastest possible decoding times). Thus, two genes with similar DTMs correspond to the same speed-budget.

In the case where *w*_1_ = … = *w*_*K*_ = 1, the DTM is referred to as the *homogeneous DTM*. A monotone-increasing weights describes the hypothesis that slower codons toward the 3’ UTR increase ribosomal “traffic jams” on the mRNA, resulting in larger number of ribosomes on the mRNA at steady-state (see S2 Fig).

### Mutated GFP genes

The GFP protein sequence is from gi:1543069. Recall that the GFP gene ORF consists of 239 codons (excluding the stop codon). The mutated GFP genes are generated by performing the following synonymous substitutions relative to the GFP gene (see also S3 Fig):

1. **GFP_HIGH_RD**: the first 119 codons (corresponding to λ_0_, …, λ_11_) are synonymously substituted with *fast* codons, and codons 120–239 (corresponding to λ_12_, …, λ_23_) are synonymously substituted with *slow* codons. This is done to create a “traffic jam” in this gene, where the total number of ribosomes along the mRNA at steady-state is high.
2. **GFP_LOW_RD**: the first 119 codons (corresponding to λ_0_, …, λ_11_) are synonymously substituted with *slow* codons, and codons 120–239 (corresponding to λ_12_, …, λ_23_) are synonymously substituted with *fast* codons. This is done to create a “traffic flow” in this gene, where the total number of ribosomes along the mRNA at steady-state is low.
3. **GFP_MDN_RD**: codons 1–79 (corresponding to λ_0_, …, λ_7_) are synonymously substituted with *slow* codons, codons 80–159 (corresponding to λ_8_, …, λ_15_) are synonymously substituted with *median* codons (i.e. in each synonymous group, the codon with the median decoding time is chosen), and codons 160–239 (corresponding to λ_16_, …, λ_23_) are synonymously substituted with *fast* codons. This is done to create a more of a subtle “traffic flow”.
4. **GFP_SPD_TR**: all codons are synonymously substituted with *fast* codons. This is done to create “fast traffic” in this gene, where the steady-state translation rate is maximal.
5. **GFP_SLW_TR**: all codons are synonymously substituted with *slow* codons. This is done to create “slow traffic” in this gene, where the steady-state translation rate is minimal.

## Supporting information

S1 Table Lists the 770 *S. cerevisiae* cell cycle related genes used in our model. (XLSX)

S2 Table Lists the codon decoding times. (PDF)

S1 Fig Depicts the parameter *c* in *G*(*z*) ≔ tanh(*z*/*c*). (PDF)

S2 FigDepicts the monotone-increasing weights in $\tilde{\eta }$.(PDF)

S3 Fig Depicts the RFMIO rates and steady-state densities of the GFP gene and its mutations. (PDF)

S4 Fig Depicts the histogram of *S. cerevisiae* ORFs codon length *K*. (PDF)

S5 FigDepicts the histograms of $R_{a}^{i}$ and $\rho_{a}^{i}$ over all *S. cerevisiae* genes for *A* = 0.35, *T* = 16, ${\tilde{L}}_{h}=20\%$, *α* = 0.8, and $\bar{z}=30\%$.(PDF)

S6 Fig Depicts the effect of heterologous gene mRNA levels and initiation rates in the presence of oscillation noise. (PDF)
