# Correction: Computational analysis of the oscillatory behavior at the translation level induced by mRNA levels oscillations due to finite intracellular resources

**DOI:** 10.1371/journal.pcbi.1007192

**Published:** 2019-07-02

**Authors:** Yoram Zarai, Tamir Tuller

In the funding statement the following sentence was omitted:

YZ also gratefully acknowledges the support of the Israeli Ministry of Science, Technology and Space (http://archive.most.gov.il/English/Pages/default.aspx).
