# Supplementary material for: Computational analysis of the oscillatory behavior at the translation level induced by mRNA levels oscillations due to finite intracellular resources
Source: PLoS Comput Biol. 2018 Apr 3;14(4):e1006055. doi: 10.1371/journal.pcbi.1006055 (PMC5898785; doi:10.1371/journal.pcbi.1006055)
Supplement: S2 Table — (PDF) [file pcbi.1006055.s002.pdf]

Table S2
CODONS DECODING TIMES IN SECONDS UNITS ("DT" DENOTES DECODING TIME).

Codon	DT	Codon	DT	Codon	DT	Codon	DT
AAA	0.1685	CAA	0.1457	GAA	0.1333	TAA	0
AAC	0.1595	CAC	0.1378	GAC	0.1392	TAC	0.1318
AAG	0.1291	CAG	0.1936	GAG	0.1553	TAG	0
AAT	0.1872	CAT	0.1750	GAT	0.1608	TAT	0.1708
ACA	0.1774	CCA	0.1674	GCA	0.1555	TCA	0.1750
ACC	0.1402	CCC	0.1972	GCC	0.1219	TCC	0.1522
ACG	0.1930	CCG	0.1960	GCG	0.1431	TCG	0.1772
ACT	0.1562	CCT	0.1870	GCT	0.1226	TCT	0.1583
AGA	0.1128	CGA	0.2078	GGA	0.1816	TGA	0
AGC	0.1581	CGC	0.1640	GGC	0.1518	TGC	0.1791
AGG	0.1444	CGG	0.2246	GGG	0.1698	TGG	0.1307
AGT	0.1626	CGT	0.1258	GGT	0.1249	TGT	0.1511
ATA	0.1725	CTA	0.1541	GTA	0.1564	TTA	0.1552
ATC	0.1349	CTC	0.1697	GTC	0.0831	TTC	0.1275
ATG	0.1263	CTG	0.1732	GTG	0.1534	TTG	0.1401
ATT	0.1549	CTT	0.1706	GTT	0.0973	TTT	0.1609
