# Supplementary material for: Computational analysis of the oscillatory behavior at the translation level induced by mRNA levels oscillations due to finite intracellular resources
Source: PLoS Comput Biol. 2018 Apr 3;14(4):e1006055. doi: 10.1371/journal.pcbi.1006055 (PMC5898785; doi:10.1371/journal.pcbi.1006055)
Supplement: S1 Fig — (PDF) [file pcbi.1006055.s003.pdf]

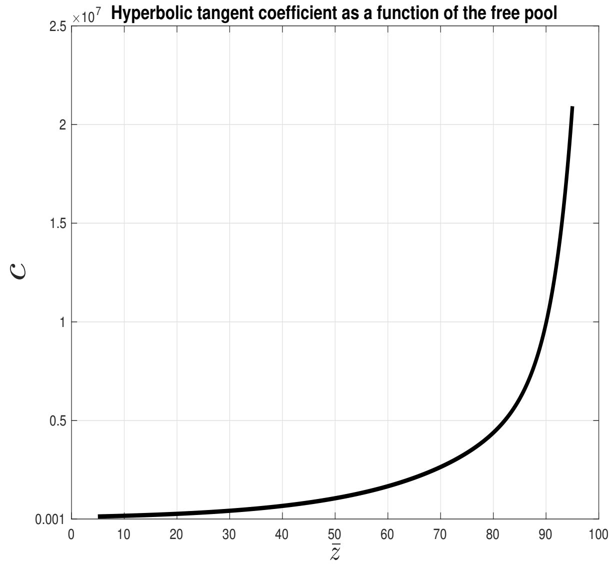

Fig. S1. The value of c in $G(z) := \tanh(z/c)$, as a function of the steady-state average free pool in percentage of the total pool. In the simulations $G^j(z) := \tanh(z/c)$, $c > 0$, is used for all $j \in \{1, \ldots, m\}$. This corresponds to the assumption of equal spatial availability of ribosomes relative to mRNA positions in the cell.
