# Supplementary figures and images for: Computational analysis of the oscillatory behavior at the translation level induced by mRNA levels oscillations due to finite intracellular resources
Source: PLoS Comput Biol. 2018 Apr 3;14(4):e1006055. doi: 10.1371/journal.pcbi.1006055 (PMC5898785; doi:10.1371/journal.pcbi.1006055)

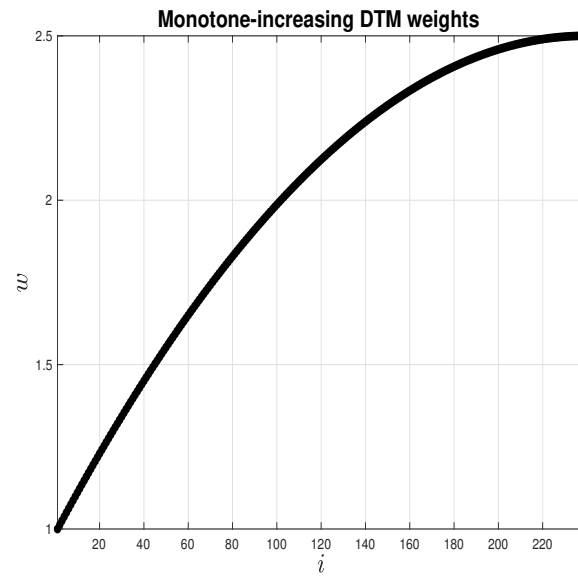

Fig. S2. DTM weights  $w_i$ s used in the computation of  $\tilde{\eta}$ .

Supplement: S2 Fig — (PDF) [file pcbi.1006055.s004.pdf]

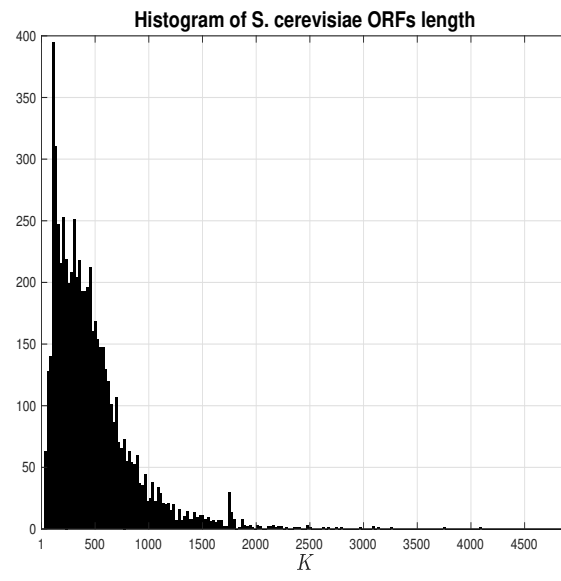

Fig. S4. Histogram of *S. cerevisiae* ORFs codon length  $K$ .

Supplement: S4 Fig — (PDF) [file pcbi.1006055.s006.pdf]
