# Supplementary material for: Computational analysis of the oscillatory behavior at the translation level induced by mRNA levels oscillations due to finite intracellular resources
Source: PLoS Comput Biol. 2018 Apr 3;14(4):e1006055. doi: 10.1371/journal.pcbi.1006055 (PMC5898785; doi:10.1371/journal.pcbi.1006055)
Supplement: S3 Fig — (PDF) [file pcbi.1006055.s005.pdf]

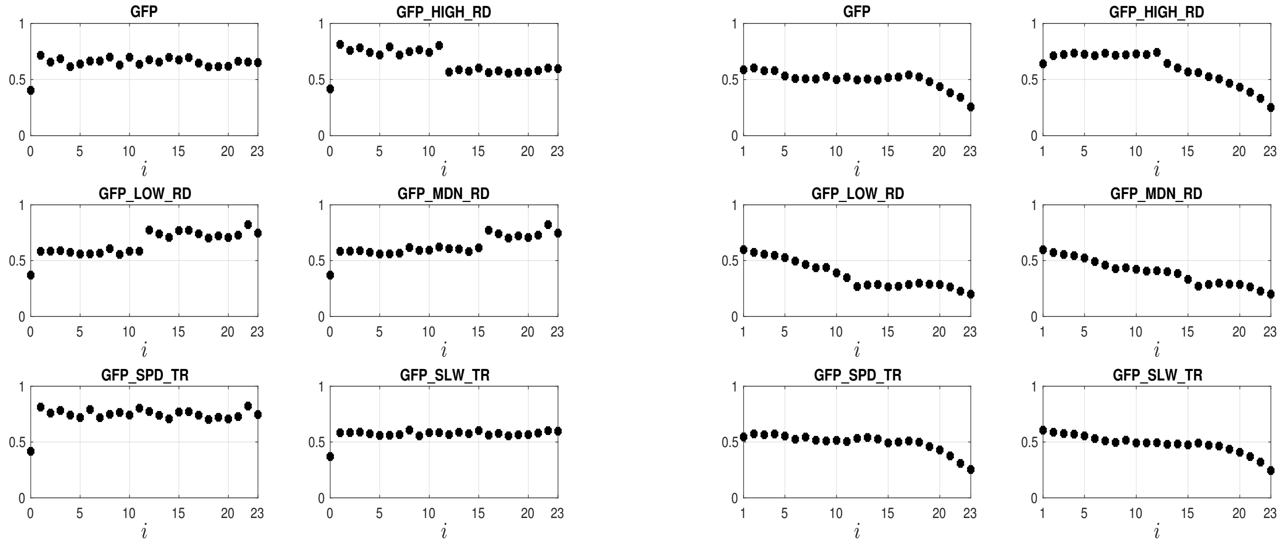

Fig. S3. RFMIO rates of the GFP gene and its mutations (left), and RFMIO steady-state densities of the GFP gene and its mutations (right).
