# Supplementary material for: Computational analysis of the oscillatory behavior at the translation level induced by mRNA levels oscillations due to finite intracellular resources
Source: PLoS Comput Biol. 2018 Apr 3;14(4):e1006055. doi: 10.1371/journal.pcbi.1006055 (PMC5898785; doi:10.1371/journal.pcbi.1006055)
Supplement: S5 Fig — (PDF) [file pcbi.1006055.s007.pdf]

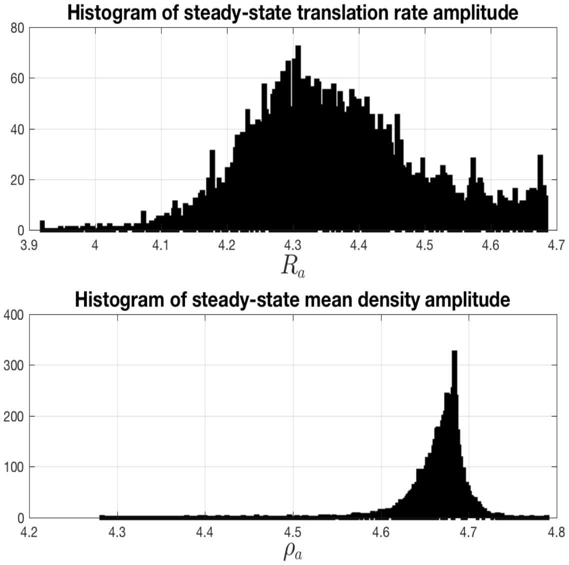

Fig. S5. Histograms of R_a^i (upper figure) and ρ_a^i (lower figure) over all *S. cerevisiae* genes for $A = 0.35$, $T = 16$, $\tilde{L}_h = 20\%$, $\alpha = 0.8$, and $\bar{z} = 30\%$. In this case, $z_a = 5.4706\%$, $\bar{R}_a = 4.3655\%$, $\bar{\rho}_a = 4.6672\%$, $\hat{R}_a = 0.0170\%$, and $\hat{\rho}_a = 0.0008\%$ (all numbers are to four digit accuracy).
