# Supplementary material for: Computational analysis of the oscillatory behavior at the translation level induced by mRNA levels oscillations due to finite intracellular resources
Source: PLoS Comput Biol. 2018 Apr 3;14(4):e1006055. doi: 10.1371/journal.pcbi.1006055 (PMC5898785; doi:10.1371/journal.pcbi.1006055)
Supplement: S6 Fig — (PDF) [file pcbi.1006055.s008.pdf]

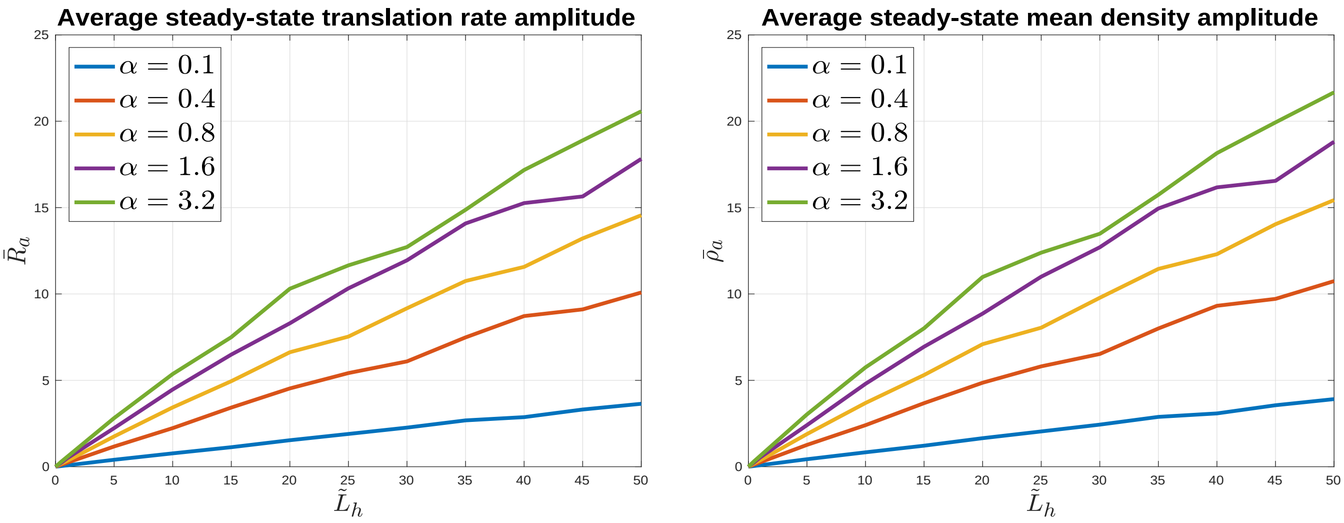

(a) (b)

Fig. S6: The effect of heterologous gene mRNA levels and initiation rates in the presence of oscillation noise. In this case, the mRNA levels of the oscillating gene are fluctuated as follows: $\ell_h(t) = L_h \left(1 + A\sin(\frac{2\pi t}{T} + w(t))\right)$, where $w(t) \sim N(0, 1/2)$ for all $t \geq 0$. (a) $\bar{R}_a$ as a function of $\tilde{L}_h$ and different values of α, for $A = 1/2$, $T = 16$, and $\bar{z} = 30\%$. (b) $\bar{\rho}_a$ as a function of $\tilde{L}_h$ and different values of α, for $A = 1/2$, $T = 16$, and $\bar{z} = 30\%$. Comparing these results to the results depicted in Fig. 3, panels (a) and (b) in the main text, respectively, (that depict the results without noise - see Eq. (1) in the main text), it can be observed that the impact of the oscillation noise on the average steady-state translation rate and mean density amplitudes is low. The same behavior can be deduced from both figures and the actual parameter values are very close. This make sense as these parameters average amplitudes over all genes.
